# Food for teens: how social media is associated with adolescent eating outcomes

**DOI:** 10.1017/S1368980021003116

**Published:** 2022-02

**Authors:** Yara Qutteina, Lotte Hallez, Maxime Raedschelders, Charlotte De Backer, Tim Smits

**Affiliations:** 1 Institute for Media Studies, KU Leuven, Parkstraat 45, P.O. Box 3603, Leuven 3000, Belgium; 2 Department of Communication Studies, University of Antwerp, Sint Jacobstraat 2, Antwerp 2000, Belgium

**Keywords:** Adolescents, Social media, Eating, Food marketing, Norms, Food literacy

## Abstract

**Objective:**

To assess the relationship between exposure to social media food messages and self-reported adolescent eating outcomes (including food intake, perceived norms and food literacy).

**Design:**

A cross-sectional survey was used to assess reported exposure to core and non-core food messages (including marketing messages) on social media, as well as reported food intake, perceived norms, food literacy, attitudes, self-regulation, among others.

**Setting:**

18 secondary schools across Flanders, Belgium.

**Participants:**

1002 adolescents 11–19 years of age.

**Results:**

Self-reported exposure to food marketing and overall food messages on social media was positively associated with eating attitudes, behaviours, perceived norms and food literacy among adolescents. Interestingly, the relationship between food exposure and intake was shaped differently depending on food type; descriptive norms mediated the positive relationship between non-core food social media exposure and non-core food intake (e.g. indirect effect estimate on self-reported sweets consumption is 0·005, se 0·002, *P* < 0·01), while food literacy mediated the positive relationship between core food social media exposure and core food intake (e.g. indirect effect estimate on self-reported vegetable consumption is 0·01, se 0·003, *P* < 0·000).

**Conclusions:**

This study highlights the significance of social media in relation to adolescent eating. There is an opportunity for health professionals to use social media in the promotion of core food among adolescents. We call for relevant policy actions to regulate the marketing of non-core food to adolescents on social media.

Most countries have been witnessing a concerning rise in adolescent obesity^(1)^. This can partly be attributed to unhealthy dietary patterns seen among adolescents. Their daily diets are characterised by high intakes of non-core foods, defined as foods dense in energy and low in nutrients, such as sugary and savoury snacks^(1,2)^. At the same time, adolescents fail to consume the recommended amounts of core foods, defined as high nutrient foods belonging to the main food groups promoted by dietary guidelines such as fruits and vegetables^(2,3)^. It is therefore no surprise that the number of obese children and adolescents worldwide has increased from 11 to 124 million in the past 4 decades only^(4)^. This is worrisome as an obese adolescent grows to have a higher risk of mortality and morbidity^(5)^ and maintain their obesity risk factors, for example, low food literacy, unhealthy dietary attitudes, habits and behaviours, in adulthood^(6)^.

One of the most important factors contributing to unhealthy dietary attitudes and eating is the obesogenic environment we live in, an environment saturated with messages encouraging non-core food consumption and promoting obesity. Food messages – such as social media images and videos, advertisements, food influencer posts, cooking shows or peer-to-peer messages – have infiltrated our environments. These messages, both virtual and non-virtual, often promote unhealthy non-core food norms^(7,8)^ celebrating non-core food intake and encouraging excess energy consumption^(7,9–12)^. A significant portion of such food messages consists of food marketing, that is, messages about branded food products, such as food advertisements and sponsorships. Evidence (particularly concerning television) shows that food marketing causes adolescents to prefer, purchase and consume non-core food^(13)^.

Nowadays, adolescents are increasingly shifting from traditional media (e.g. television and magazines) to digital and social media^(14)^. For example, in Belgium, the percentage of Flemish adolescents who report daily use of live television dropped from 37·7 % in 2015 to 22 % in 2020; at the same time, 98 % of adolescents reported using social media in 2020^(15)^. Similar to the real-world and traditional media, marketed food messages comprise a large portion of the food messages on digital media^(16)^. Marketers are increasingly shifting their focus to food marketing on digital media, including social media^(14)^. On digital and social media, marketers benefit from using three main marketing strategies including paid media marketing (e.g. paid advertising, sponsorships or product placement in-exchange for a payment^(17)^), owned media marketing (defined as marketing on the brand’s owned asset^(17)^; e.g. promoting one’s brand on one’s own webpage) and earned media marketing (also known as word-of-mouth marketing and defined as marketing by the consumer without any monetary exchange^(17)^, e.g. when peers recommend branded food products without receiving a payment for this promotion). Earned media marketing is flourishing on social media where users are allowed to generate their own content and share other’s messages. Considering the popularity of social media among adolescents, it is no surprise that this age group is highly exposed to (branded) food messages online, mostly promoting the consumption of non-core foods^(11,18)^.

Beyond food marketing, adolescents are also regularly and heavily exposed to posts from peers and these often refer to food, such as influencers drinking soft drinks, or peers praising fast-food chains^(11)^. These food posts also convey food norms on adolescents’ social media networks. High exposure to such food images could easily influence perceptions about what foods others typically eat (also known as descriptive norms^(19)^), or what foods they should eat (also known as injunctive norms^(19)^). Unfortunately, the food messages that adolescents mostly see on social media are posts – primarily shared by marketers, influencers and friends – containing large quantities of non-core foods^(11)^. These social media messages may influence adolescents’ norm perceptions and make them believe that overconsumption of non-core food is the norm. Adolescents indeed have a tendency to overestimate their peers’ favourable attitude towards and intake of non-core foods while they underestimate their peers’ favourable attitudes towards and intake of core foods^(20)^. This is worrisome considering that food norm perceptions are a powerful determinant of food intake^(20,21)^. Accordingly, it is important to understand the effect of those abundant descriptive and injunctive norms favouring non-core food on social media. One would expect that exposure to such norms virtually on social media, in the same way as non-virtual norm exposure, leads adolescents to eat more of the foods they see on their social networks. Accordingly, the foods that adolescents see on social media could become norms that mediate the relationship between exposure to food messages and adolescent eating. In other words, social media food messages favouring non-core food could lead to increased positive perceptions of descriptive and injunctive non-core food norms among adolescents, which in turn, encourages them to select and eat non-core food.

In addition to norms, adolescents’ response to food messages partially depends on the practical knowledge, attitudes and skills they possess about food, which is commonly referred to as food literacy^(22)^. Vidgen and Gallegos (p. 54) define food literacy to include ‘a collection of inter-related knowledge, attitude and behaviours required to plan, manage, select, prepare and eat foods to meet needs and determine intake’^(23)^. Such food literacy skills are important in empowering adolescents to achieve better eating outcomes^(24)^. These necessary skills also direct adolescent’s food attitudes, inform their food decisions and influence their food consumption^(24,25)^. However, how is one’s food literacy shaped? The food-related content that adolescents encounter online is an important source of information that can either help or distort their food literacy^(26)^. As such, higher exposure to core food messages, such as core food recipes and core food nutrition facts, may improve adolescents’ food literacy. Furthermore, many consider enhancing food literacy as an important approach in changing eating behaviour towards increased core food consumption^(26,27)^. In other words, core food content on social media could increase food literacy, and a raised level of food literacy could lead to increased intakes of core food^(28)^. Accordingly, higher food literacy may mediate core food intake and act as a buffer against messages promoting non-core foods.

Despite the known risks of media and marketed food messages on eating, particularly among adolescents, there is a dearth of research investigating the link between media food messages and adolescents’ eating. Over the years, scholars have investigated the effect of food marketing on food consumption, yet most research has focused on traditional media marketing targeting adults or young children under 12 years of age^(9,13)^. Very few studies have researched food marketing targeting adolescents older than 12 years of age^(13)^. Adolescence is a complex lifestage that necessitates its own special focus. Adolescents increasingly make choices independent from their parents^(29)^; they experience and exert more peer influence^(30)^ and are exposed to more (social) media and advertising compared with younger children^(18,31)^. Furthermore, adolescents are particularly susceptible to the influence of unhealthy food norms on social media^(31,32)^. This is due to the larger social networks they live in compared with children^(30)^ and an increased use of social media (together with a decrease in parental mediation of this media consumption^(29)^). Consequently, an adolescent is influenced more by external factors including the norms set by the media and food marketing as compared with children under 11 years of age^(29)^. It is therefore paramount to understand how social media food messages, including marketed messages, influence adolescents’ eating habits. A systematic review and meta-analysis by Qutteina *et al.* found that the little research examining media food marketing’s effect on adolescents already shows evidence of a small effect size on eating outcomes^(13)^. This review also highlighted a lack of studies on social media food marketing, especially during late adolescence 14–18 years of age, despite adolescents’ high use of social media^(13,15)^.

In sum, this exploratory study focuses on this group of adolescents for which there is a dearth of evidence concerning the relation between their social media behaviour and food attitudes and consumption. The study aims to answer the following main research questions pertaining to both core foods and non-core foods:

RQ1: What is the relationship between self-reported exposure to social media food messages and eating outcomes (including attitudes, perceived norms, food literacy and self-reported food intake) among adolescents 11–19 years old?

In addition, we also aim to explore the underpinnings of this relation, zooming in on the potential mediating role of both perceived social norms and food literacy:

RQ2a: How do perceived social norms mediate the relationship between self-reported social media exposure to core and non-core food messages and self-reported core and non-core food intake among adolescents?

RQ2b: How does food literacy mediate the relationship between self-reported social media exposure to core and non-core food messages and self-reported core and non-core food intake among adolescents?

## Methods

To assess the relationship between social media food message exposure and adolescent eating, a cross-sectional survey was carried out across secondary schools in Belgium. The research protocol was reviewed and approved by the first author’s university’s board for ethical review (file G-2018 06 1257).

### Sample

Adolescents aged 11–19 years, attending schools in Flanders, were randomly selected based on a multistage cluster sampling. First, schools were randomly selected from the official list of the public and private secondary schools provided by the Ministry of Education in Flanders. The sample of schools was checked to guarantee that all school types (general, technical, professional) were sufficiently represented. The selected schools were oversampled by 78 % to account for low response rates among Flemish schools^(33)^. The sample excluded adolescents who did not speak Dutch, who were above 19 years of age or below 11 years of age. Initially, we aimed for a sample of 12–19-year-old adolescents. However, the age range was extended to include some 11-year-old adolescents (*n* 13) who completed the questionnaire alongside their classmates.

### Recruitment

Each selected school received recruitment emails and was contacted via phone by members of the research team. A total of eighteen schools agreed to participate and provided access to a minimum of two classes each. The to-be expected sample size, based on the participation of these schools and based on the linear regression rule-of-thumb principles of fixed sample size or subjects-per-variable, was deemed sufficient^(34)^. Well above 1000 participants were expected to be sampled, from a varied background. 1232 adolescent participants (or their parents) were contacted for recruitment.

Following the guidelines of the ethical review board, parental consent was required from adolescents under 16 years of age prior to their participation in the study. The schools facilitated this active parental consent procedure, by sending parents emails with a link to the digital parental consent form or by providing hard copies of the parental consent forms which were signed by the parents and returned back to the schools and researchers. Over 76 % of the contacted parents, of adolescents younger than 16 years, actively consented for their child to participate. Following the attainment of parental consent (when applicable), the schools were visited by one or two researchers. Three trained researchers, individually or in groups of two, carried out data collection. In classroom settings, the researchers introduced the study to participants and orally explained the consent/assent forms, before providing them a link to the online survey. Adolescent participants were seated as far apart as possible from each other and were asked to work quietly and individually. Depending on the availability of computers and tablets in school, participants could access the survey via computer, tablet or their own smartphone device. The first page of the online survey was a digital informed consent/assent form. For those who did not consent/assent or whose parents did not provide parental consent (when needed), a digital assignment was provided to help them fill the time and avoid any possible coercion in recruitment. The researchers remained in the classroom, available to answer any question or concern participants may have had regarding the study.

### Materials

In-depth semi-structured cognitive interviews were conducted prior to the study to assess the face validity of several sections of the questionnaire including food marketing exposure, norms, attitudes, intentions and self-treatment regulation. Based on the cognitive interview results, the questions were adapted when deemed necessary. The survey instrument was programmed into Qualtrics^(35)^ and piloted among 300 Flemish adolescents. Researchers administered the questionnaire during class hours at participating secondary schools.

Following are the variables included in the survey (also see online supplementary material, Supplemental Table 2). Several scales (excluding food intake) assessed a measurement in reference to core or non-core food. Foods that belong to the dietary guidelines main food groups, and that were coded as core foods following the classifications of Toumpakari *et al.* and Kellet *et al.*, included water and unsweetened drinks, fruits and vegetables^(2,36)^. Foods that are energy dense, low-nutrient and do not belong to any of the dietary guidelines main food groups were coded as non-core foods and included sweetened drinks, sweets and salty/savoury snacks^(2,36)^.

#### Food messages exposure

The exposure outcome was measured via thirty-five items that inquired about the extent to which the participant saw core and non-core food messages on their social media. Participants reported the extent to which they saw food messages posted by friends, influencers and celebrities as well as messages posted by brands, on a 5-point scale ranging from ‘not at all’ to ‘very often’.

#### Food intake

Intake was measured via a Flemish Food Frequency Questionnaire that inquired about the frequency and portion of individual food items, ranging between core (e.g. water, vegetables and fruits) and non-core (e.g. soft drinks, fried food, chips and candy) foods, consumed during the past month^(37)^. Participants reported, on a 6-point Likert scale, the consumption frequency (never, 1–3 d/month, 1 d/week, 2–4 d/week, 5–6 d/week or daily) of food items included in this study excluding water. For water and other food items (excluding fried food) that were consumed during the past month, participants were asked to indicate the amounts of food consumed per day on a 4-point Likert scale. The scales differed depending on the food items measured. Participants reported their water intake on a scale ranging from 500 ml or less to more than 1250 ml, sugared drinks intake on a scale ranging from 250 ml or less to more than 750 ml, sweet and salty non-core food snack intake on scales ranging from 50 g or less to more than 100 g, fruit intake on a scale ranging from 150 g or less to more than 450 g and vegetable intake on a scale ranging from 60 g or less to more than 300 g.

#### Intention to eat

Adolescents’ intentions to eat core and non-core foods were also measured by asking participants to think about the following month, and to indicate how much food they plan to eat, compared with what they eat now^(38)^. Respondents chose from a 5-point scale ranging from ‘much less’ to ‘much more’.

#### Food attitudes

Food preferences and perception of food healthiness were used to measure core and non-core food attitudes. Both scales were adapted from Dixon and Colleagues^(38)^. Food preferences were assessed by the question ‘How much do you like each of these foods?’, and participants chose an answer from a 5-point Likert scale that ranged from 1 ‘Hate it’ to 5 ‘love it’. Another attitude indicator, perception of healthiness, was assessed by the question ‘In your opinion, how healthy are each of these foods?’ to which respondents chose an answer on a 5-point scale from 1 ‘very unhealthy’ to 5 ‘very healthy’.

#### Perceived norms

Perceived norms were measured following the standard procedure within norm perception research, and using 5-point scale statements adapted from Dixon *et al.* ^(38)^. In this study, we differentiated between descriptive norms and injunctive norms. Descriptive norms were measured by asking participants to respond on a scale of 1 ‘rarely’ to 5 ‘very often’ to the question ‘how often do you think other children your age consume this food?’. Injunctive norms were measured by asking participants to respond on a scale of 1 ‘very unhealthy’ to 5 ‘very healthy’ to the question ‘how healthy do others think this food is?’.

#### Food literacy

The validated self-perceived food literacy scale^(39)^ was used to assess participants’ level of food literacy in several areas. The scale consisted of measures of food preparation skills, resilience and resistance (behavioural control and self-efficacy), healthy snack styles, food label examination and daily food planning. Participants responded to 5-point scales ranging from ‘no, never’ to ‘yes, always’.

#### Self-regulated autonomy

Participants’ motivation to freely engage in core eating behaviours was assessed using the validated treatment self-regulation questionnaire comprising ten items^(40,41)^.

#### BMI-for-age

Participants self-reported their weight and height measurements. These values were used to calculate their BMI-for-age in accordance with the WHO Child Growth Standards^(42)^.

#### Demographics

Participants were also asked demographic questions including gender, age and indicators of socio-economic status (SES) including mother’s highest level of education^(43)^ and school education type. In Belgium, the type of school education an adolescent is enrolled in -classical (theoretical), vocational or professional- is typically used as an indication of SES. However, in Belgium’s educational system only 8th–12th graders follow this type of educational categorisation.

#### Analysis

Data analysis was conducted in R software 2019 (R version 3.6.1)^(44)^. The data were first cleaned to remove invalid responses (e.g. illogical responses in open-ended questions), straightlined responses (Careless package was used to remove same-value responses given consecutively over a long string of items^(45)^) and questionnaires completed in a very short and unrealistic amount of time. *T*-tests and Cohen’s d were used to assess the difference in attitudes and perceived norms scores favouring core *v*. non-core food. Kruskal–Wallis test was used to assess the difference in median scores of self-reported exposure to core, non-core and branded non-core food messages on social media. Kendal’s rank correlation was used to assess bivariate relationships between reported exposure to social media food messages and the different eating outcomes, that is, food intake, attitudes, perceived norms and food literacy, whereby all analysis assumptions were met.

To analyse the relationship between reported exposure to social media food messages, food intake, perceived norms and food literacy, mediation models were analysed with structural equation modelling – specifically path analysis – using the Lavaan package^(46)^. The analysis was repeated using ordinary least square regressions models in Process Hayes for R^(47)^. Both statistical methods are commonly used in such analyses^(48)^ and can handle multiple mediators and outcomes. The mediation models included exposure to core food posts, non-core food posts and branded food posts as exposure variables, and intake of core and non-core food as outcome variables. The models included perceived norms and food literacy as mediators, and controlled for gender, age, BMI-for-age and self-regulated autonomy as covariates. The models were found to have a proper fit after the removal of self-regulated autonomy and injunctive norms; however, this did not change the results or significance of the models (see online supplementary material. Supplemental Table 7). For theoretical comprehensiveness, the models with self-regulated autonomy and injunctive norms will be presented in this paper.

SES indicators were initially included in the analysis, but were later removed because their addition reduced the sample size without changing the models. Mother’s educational attainment (as an indicator of SES) was initially inserted in the models as a covariate. Considering that this addition did not change the model results and that more than 25 % of the participants did not know their mother’s educational attainment, this SES indicator was later dropped. The models were also performed exclusively for 3rd–6th graders (aged 13–19 years) with school education type (as an indicator of SES) inserted as a covariate. However, the inclusion of this SES indicator did not improve the models, and it also was dropped from the analysis.

## Results

A total of 1098 Flemish adolescents between the ages of 11–19 from schools across Flanders, Belgium participated in the study. Following cleaning, a total of 1002 (M = 15, sd = 2·06) participants were included in the study. Sample respondents included 58 % female and 42 % male adolescents. Further descriptive characteristics are presented in Table 1. Based on *t*-test analyses, it seems that adolescents reported significantly higher preferences and perceived descriptive norms for non-core food compared with core food. On the other hand, adolescent participants showed significantly higher perceived healthiness of and injunctive norms favouring core food (see Table 2). Furthermore, the participants reported significantly (H(70) =315·94, *P* < 0·000) higher exposure to non-core food messages (Mdn = 4·08) compared with core food messages (Mdn = 2·58). Adolescents also reported significantly (H(18) = 131·00, *P* < 0·000) higher exposure to branded non-core food messages (Mdn = 5·00) compared with overall core food messages. Further descriptive details on the participants’ reported exposure to social media food messages and reported food intake can be found in online supplementary material, Supplemental Tables 3–6.

**Table 1:** Characteristics (age, gender, educational attainment and mother’s educational attainment) of study sample of 1002 adolescent participants

Sample characteristics	*n*	%
Age		
11–15	543	54
16–19	459	46
Gender		
Male	422	42
Female	579	58
School education type*		
General education	70	12
Technical education	292	48
Vocational education	243	40
Art education	3	0
Mother’s educational attainment		
Less than Secondary degree	63	6
Secondary degree	206	21
College degree	263	26
University degree	193	19
Don’t know	247	25

* Only for adolescents in 8th–12th grade (13–19 year olds) in secondary school.

**Table 2:** Mean scores and standard deviations of reported food attitudes, perceived food norms and food literacy among 1002 Flemish adolescents (11–19 years old), and the differences between these scores in relation to core and non-core food

Variable	Mean	sd	Difference between core and non-core foods	Cohen’s d	95 % CI
Food preferences					
Core food	3·67	0·82	*t* (1942·1) = -9·53	0·43	0·31, 0·55
Non-core food	4·03	0·88			
Perceived healthiness					
Core food	4·35	0·78	*t* (1975·9) = 83·14	3·73	3·46, 4·00
Non-core food	1·53	0·73			
Perceived descriptive norms					
Core food	2·92	0·81	*t* (1963·1) = -23·74	1·07	0·93, 1·21
Non-core food	3·81	0·86			
Perceived injunctive norms					
Core food	4·54	1·18	*t* (1868·1) = 49·94	2·26	2·06, 2·45
Non-core food	2·12	0·95			
Food literacy	3·31	0·48	NA	NA	NA

### Social media food messages are associated with eating attitudes and behaviours

Using Kendal’s rank correlation tests, self-reported exposure to food messages on social media was found to be associated with eating attitudes and self-reported eating behaviours among adolescents. The more they reported seeing non-core food posts, the more they reported higher preference and intake of non-core food (*Z* = 3·630, *P* < 0·000). After controlling for age, gender, BMI-for-age, self-regulated autonomy, perceived norms and food literacy, the mediation model showed self-reported exposure to non-core food messages on social media still was significantly associated with increased sweet and fried food intake (see online supplementary material, Supplemental Table 3 and Table 8).

When focusing exclusively on food marketing on social media, the trend seen with non-core food messages seems to extend to include branded posts as well. Adolescents who reported more exposure to social media food marketing, particularly non-core food marketing, were significantly more likely to report non-core food preferences (*Z* = 3·388, *P* < 0·000).

These findings partially answer RQ1, revealing that exposure to different types of social media food messages is associated with different eating outcomes. Increased exposure to non-core food messages, including non-core food marketing, is associated with increased preferences (attitudes) and intake of non-core food. To fully answer RQ1, we look at the relationship between social media food exposure and perceived norms and food literacy in the following sections.

### Social media food messages are associated with perceived food norms

Adolescents who reported higher exposure to social media messages portraying non-core food seemed to report higher perceived descriptive norms favouring non-core food, while those who reported exposure to more core food messages seemed to report higher perceived descriptive norms of core food. Overall, *t*-tests demonstrated that adolescents reported significantly higher perceived descriptive norms of non-core food compared with core food (*t* = 23·74, *P* < 0·000), meaning they perceive their peers to especially consume non-core foods (see Table 2). Furthermore, Kendall’s rank correlation tests showed that adolescents perceived higher descriptive norms favouring non-core food when exposed to more social media messages of non-core food (*Z* = 5·626, *P* < 0·000). Adolescents exposed to higher social media food marketing, particularly non-core food marketing, seemed to believe that others highly consume non-core food as they reported higher non-core food descriptive norms (*Z* = 5·333, *P* < 0·000). On the other hand, adolescents who reported higher exposure to core food posts were more likely to believe that others highly consumed core food as they reported higher descriptive norms favouring core food (*Z* = 2·931, *P* < 0·01). These results partially answer RQ1 and show that self-reported exposure to social media food messages is significantly associated with perceived norms, particularly descriptive norms, among adolescents.

**Fig. 1:**
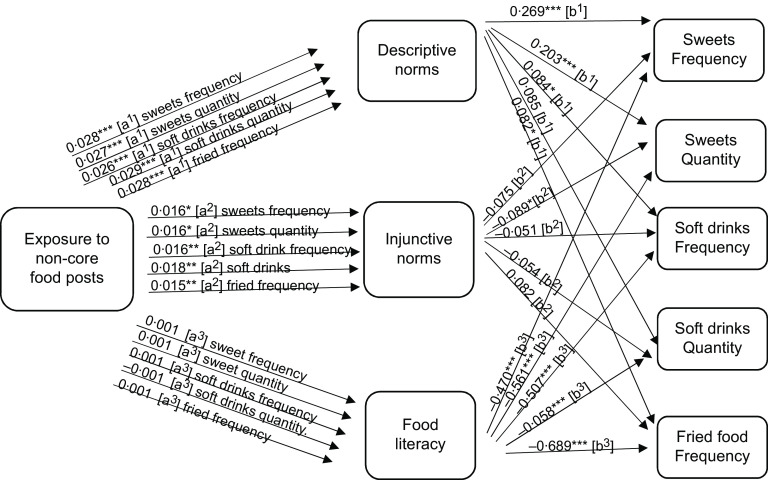
Path analysis mediation model showing the indirect effects (a and b pathways) of reported exposure to non-core food social media posts on self-reported non-core food consumption (measured as frequency and quantity per month) among Flemish adolescents 11–19 years old. Significance **P < 0·05, ***P < 0·01, ****P < 0·000

**Fig. 2:**
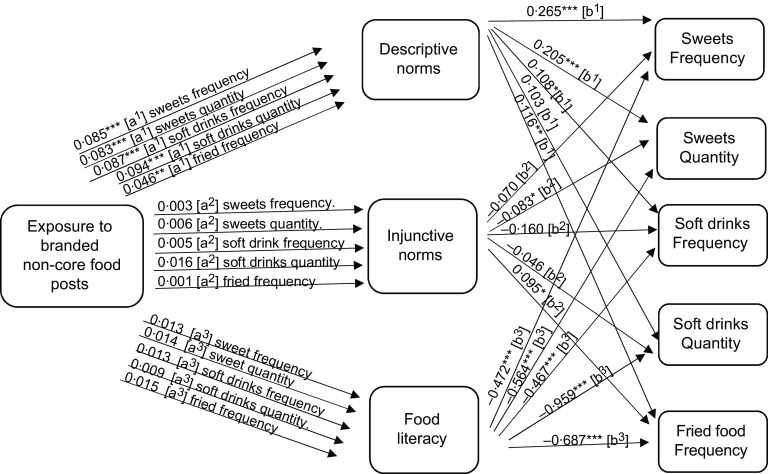
Path analysis mediation model showing the indirect effects (a and b pathways) of reported exposure to branded non-core food social media messages on self-reported non-core food consumption (measured as frequency and quantity per month) among Flemish adolescents 11–19 years old. Significance **P < 0·05, ***P < 0·01, ****P < 0·000

To answer RQ2a, mediation analysis was performed to focus on the potential mediating role of perceived norms. Descriptive norms mediated the relationship between exposure to non-core food posts (as well as branded non-core food posts) on social media and adolescent eating (see the indirect effects in Figs. 1–2, and the model results in Tables 3–4, and online supplementary material, Supplemental Tables 8–10).

**Table 3:** Mediation models demonstrating the relationship between reported exposure to non-core food posts on social media and reported non-core food intake among Flemish adolescents 11–19 years old

	Sweets (Frequency)			Sweets (quantity g/d)			Soft drinks (Frequency)			Soft drinks (quantity ml/d)			Fried food (Frequency)		
	Effect Estimate	se	*P*	Effect Estimate	se	*P*	Effect Estimate	se	*P*	Effect Estimate	se	*P*	Effect Estimate	se	*P*
Direct effect of non-core food posts	0·015	0·006	0·022	0·015	0·006	0·022	0·007	0·008	0·388	0·013	0·009	0·145	0·033	0·007	0·000
Indirect effects															
Descriptive norms	0·005	0·002	0·001	0·005	0·002	0·001	0·003	0·001	0·033	0·002	0·002	0·140	0·002	0·001	0·065
Injunctive norms	−0·001	0·001	0·082	−0·001	0·001	0·072	−0·001	0·001	0·220	−0·001	0·001	0·327	0·001	0·001	0·113
Food literacy	−0·001	0·002	0·725	−0·001	0·002	0·725	−0·000	0·002	0·800	0·001	0·002	0·755	−0·001	0·002	0·776
Total indirect	0·003	0·003	0·175	0·001	0·003	0·185	0·002	0·002	0·483	0·003	0·002	0·490	0·003	0·003	0·257
Total effect	0·011	0·008	0.007	0·018	0·007	0·007	0·008	0·008	0·292	0·015	0·008	0·094	0·036	0·008	0·000

**Table 4:** Mediation models demonstrating the relationship between branded non-core food posts on social media and non-core food intake among Flemish adolescents 11–19 years old

	Sweets (frequency)			Sweets (quantity g/d)			Soft drinks (frequency)			Soft drinks (quantity ml/d)			Fried food (Frequency)		
	Effect estimate	se	*P*	Effect estimate	se	*P*	Effect estimate	se	*P*	Effect estimate	se	*P*	Effect estimate	se	*P*
Direct effect of branded non-core food posts	0·024	0·023	0·308	0·034	0·021	0·101	−0·008	0·024	0·724	0·000	0·009	0·990	0·030	0·017	0·168
Indirect effect															
Descriptive norms	0·023	0·006	0·000	0·017	0·005	0·001	0·009	0·004	0·023	0·010	0·006	0·081	0·010	0·004	0·014
Injunctive norms	−0·000	0·002	0·760	−0·001	0·002	0·727	−0·000	0·001	0·763	−0·001	0·001	0·557	0·000	0·002	0·878
Food literacy	−0·006	0·005	0·216	−0·008	0·006	0·191	−0·006	0·005	0·218	−0·009	0·010	0·369	−0·009	0·007	0·223
Total indirect	0·016	0·008	0·043	0·009	0·008	0·270	0·003	0·007	0·668	−0·000	0·011	0·998	0·001	0·008	0·868
Total effect	0·040	0·024	0·097	0·043	0·022	0·048	−0·005	0·024	0·824	0·000	0·030	0·991	0·032	0·024	0·192

### Social media food messages are associated with food literacy

Kendall’s rank correlation tests showed significant association between exposure to social media food messages and food literacy on the one hand and food literacy and food intake on the other hand. Adolescents who reported lower exposure to non-core food messages on social media were significantly more likely to demonstrate higher food literacy (*Z* = -5·392, *P* < 0·000). Furthermore, food literacy was always significantly associated with self-reported food intake regardless of the type of food message adolescents reported exposure to on social media. Adolescents who scored higher on food literacy were significantly more likely to report higher core food intake (e.g. *Z* = 5·905, *P* < 0·000 with fruits, *Z* = 6·412, *P* < 0·000 with vegetables), and lower non-core food intake (e.g. *Z* = -7·072, *P* < 0·000 with soft drinks). With this finding, we have now fully answered RQ1 and found that self-reported exposure to social media food messages is associated with adolescent eating outcomes, including food literacy, perceived descriptive norms, food attitudes and food intake.

**Fig. 3:**
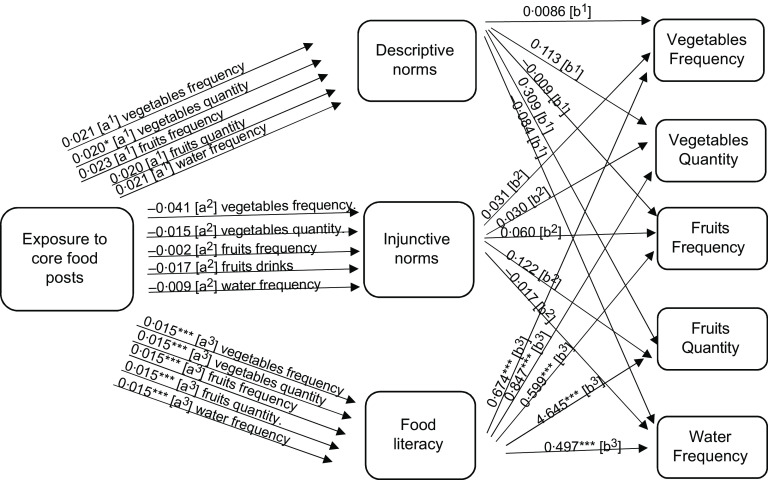
Path analysis mediation model showing the indirect effects (a and b pathways) of reported exposure to core food posts on self-reported core food consumption (measured as frequency and quantity per month) among Flemish adolescents 11–19 years old. Significance **P < 0·05, ***P < 0·01, ****P < 0·000

Next, we focus on the potential mediating roles of food literacy, as suggested in RQ2b. The mediation analysis demonstrated that food literacy mediated the relationship between reported exposure to core food posts and increased reported core food intake. However, food literacy did not mediate the relationship between reported exposure to non-core food or branded non-core food social media messages and food intake (see the indirect effects in Figs. 1–3, and the model results in Tables 3–5, and online supplementary material, Supplemental Tables 8–10). As such food literacy plays a mediating role in the core food relationships only.

**Table 5:** Mediation models demonstrating the relationship between core food posts on social media and core food intake among Flemish adolescents 11–19 years old

	Vegetables (frequency)			Vegetables (quantity g/d)			Fruits (frequency)			Fruits (quantity g/d)			Water (quantity g/d)		
	Effect estimate	se	*P*	Effect estimate	se	*P*	Effect estimate	se	*P*	Effect estimate	se	*P*	Effect estimate	se	*P*
Direct effect of core food posts	−0·011	0·009	0·219	0·011	0·009	0·226	0·005	0·008	0·548	0·040	0·043	0·359	0·006	0·009	0·489
Indirect effect															
Descriptive norms	0·002	0·001	0·100	0·002	0·001	0·072	−0·000	0·001	0·841	0·006	0·005	0·221	−0·002	0·001	0·122
Injunctive norms	−0·000	0·000	0·399	−0·000	0·001	0·447	−0·000	0·000	0·796	−0·002	0·004	0·575	0·000	0·000	0·656
Food literacy	0·010	0·003	0·000	0·013	0·003	0·000	0·009	0·002	0·000	0·068	0·018	0·000	0·008	0·002	0·000
Total indirect	0·012	0·003	0·000	0·015	0·004	0·000	0·008	0·003	0·002	0·071	0·019	0·000	0·006	0·002	0·015
Total effect	0·000	0·010	0·974	0·026	0·010	0·007	0·013	0·009	0·125	0·111	0·047	0·018	0·012	0·009	0·173

## Discussion

This study demonstrates how food messages and advertisements on social media are linked with adolescent eating. We found that adolescents who reported higher exposure to social media posts of non-core food were significantly more likely to report higher consumption of non-core food. This finding is in agreement with recent research on children’s food exposure to social media food marketing, where children were found to increase their intake of non-core food when exposed to social media messages promoting non-core food^(49)^. In this survey, branded food messages did not show a significant association with reported food intake, but this self-reported measure does not reflect the full extent of exposure to food marketing on social media. Food marketing strategies on social media are not always obvious to adolescents; therefore, the measurement of social media messages in this study included all food messages an adolescent is exposed to on these networks including marketed (branded) messages posted by peers, celebrities and influencers. In fact, previous research has demonstrated that the majority of social media food messages are marketed messages that are spread via word-of-mouth or sponsored marketed messages which influencers and celebrities are paid to promote^(11)^. Accordingly, this research adds to previous literature that found evidence of an effect by television and other traditional media marketing on adolescent eating^(13)^, by suggesting that exposure to social media marketing also is associated with adolescent eating and has comparable effects to traditional media advertising.

The findings of this study also support the notion that social media messages shape normative perceptions and that these perceptions in turn induce unhealthy eating behaviours. We found that adolescents were more likely to believe that their peers consumed more non-core food than they did core food. This finding is consistent with previous literature that found adolescents to have higher descriptive norms of unhealthy behaviours, including the consumption of non-core food, compared with healthy behaviours^(20)^. Such norms exert pressure on adolescents to conform and eat similar to the group, which is concerning especially during a lifestage where adolescents are particularly vulnerable to peer pressure^(30)^. Previous literature has provided much evidence in favour of a social norm effect on eating behaviour^(21,50)^. This study delved further and found that perceived descriptive norms (i.e. beliefs of what others eat) played a significant role in shaping the relationship between exposure to food messages and food consumption among adolescents. Perceived descriptive norms favouring non-core food mediated the relationship between reported exposure to marketed and non-marketed non-core food social media messages and reported intake of such non-core food. Injunctive norms (i.e. beliefs of what others think is healthy food), on the other hand, had no significant effect or mediating effect on adolescent food intake. Our findings align with previous studies on children and adolescents that found descriptive norms had stronger effects than injunctive norms^(51)^. This finding is also in accordance with studies that found no relationship between intake and injunctive norms when the norm was measured using more suggestive and less insisting wording (i.e. ‘encourage’ rather than ‘should ’)^(50)^. The injunctive norms in this study were worded as ‘how healthy do others think this food is’ which is very soft as compared with ‘others think I should eat this food’.

Furthermore, findings of this study lend support to recent research that supports descriptive norms as a predictor in the Theory of Planned Behaviour. Traditionally, the Theory of Planned Behaviour suggests that, among others, injunctive norms predict an adolescent’s eating intentions and behaviour^(52)^. However, research has found that injunctive norms are not as powerful in predicting behaviour as descriptive norms^(53)^, which is also a finding confirmed in this research. Findings of this research are also supportive of the social norms theory which suggests that exaggerated beliefs about non-core food consumption among peers lead to increased non-core food intake among adolescents.

The relationship between core food social media messages and intake took a somewhat different turn, as norms did not play a role in mediating this relationship. One possible explanation is that the descriptive norms favouring core food on social media were not strong enough to encourage core food consumption. Another possible explanation is that the descriptive norms on social media are mostly those that favour the consumption of non-core food rather than core food. According to a review by Stok *et al.*, descriptive norms are mostly associated with intake of foods typically eaten in friend-related social contexts^(50)^. A content analysis of social media food messages indeed indicated that non-core food messages are attached to social contexts such as hanging out with friends, whereas this was less often the case for core-food messages^(11)^. Rather, social media core-food messages were more often linked to home-cooked meals and recipes^(11)^ and may therefore play a bigger role in strengthening knowledge and practical skills concerning core foods, in turn influencing intake of those foods. In line with this reasoning, food literacy significantly mediated the relationship between reported exposure to social media core food posts and reported core food eating in this study.

This study’s findings are aligned with literature that found food literacy to be linked to both: social media messages and better core food eating outcomes^(26,54)^. Literature stresses the importance of social media as a tool to improve food literacy^(26)^. In fact, scholars refer to the food messages (e.g. food recipe videos and posts, food tips, etc.) communicated on social media as ‘social food’^(26)^. Hence, one cannot deny the significance of social media in determining an individual’s food literacy, certainly among impressionable adolescents. Furthermore, in this study, similar to a number of previous studies, increased food literacy seemed to increase core food intake. This is in accordance with literature that suggests a positive association between food literacy and core food intake. For example, a quasi-experimental study by Caraher *et al.* showed that pre-adolescents who increased their food preparation skills had higher cooking confidence and vegetable intake^(54)^. Another 10-year cohort study by Laska *et al.* found that involvement in food preparation increased consumption of fruits and vegetables and decreased non-core food intake later in adulthood^(55)^. An additional factor that this study sheds the light on is the mediation role of food literacy in the relationship between exposure to social media core food messages and core food eating. We found food literacy as the link between increased social media exposure and improved core-food intake. This stresses the importance of social media as a tool to improve both food literacy and intake.

Some study limitations should be noted. Firstly, we assessed food consumption and social media food messages and marketing exposure based on self-reported measures. As such recall bias is possible, yet was controlled for using validated and tested measurements. Other possible sources of information bias are interviewer and social desirability biases. However, we believe both biases were limited as participants completed the questionnaires by themselves without assistance from the researchers, and the researchers were present only to introduce the project and consent (or assent) participants, assure participants of the anonymity and confidentiality of their responses, ensure that standardised research protocols are followed and answer technical or clarification questions. At the same time, all researchers were trained to respond in similar, standardised manners, and without showing any partiality. Furthermore, such biases are expected to exert more influence on results of observational research (where the focus is on the basic nature of a variable) rather than correlational research (where the focus is on investigating how variables relate to each other such as in this study). Secondly, it was not possible to randomly select adolescents from the schools; however, the schools themselves were randomly selected, and the resulting sample resembles the adolescent population in Flanders, Belgium. Compared with the general Flemish adolescent population, the study’s sample had a slightly higher percentage of female to male ratio and lower SES level^(56)^. However, this study complements most studies conducted among adolescents which typically include participants of higher SES level.

Another possible limitation is the requirement of parental consent which may have limited our sample; however, every effort was made to ensure that parents received the consent form. Based on the school’s recommendations, parental consents were sent in paper, digital or both forms. Additionally, several reminders were sent to the parents before the day of the questionnaire administration. Following these measures, helped us achieve a relatively high parental consent rate. Finally, we only assessed the association between social media food messages and adolescent eating, as such it is possible that adolescents are exposed to and consume a certain food type because it is the food that fits their interest. For example, an adolescent who is more interested in non-core foods such as sweets and soft drinks will more likely consume this food type and look for it on their social media. Social media advertising, on the other hand, will also target the adolescent with advertisements that fit this adolescent’s interests, exposing them to more non-core food advertisements. Furthermore, the effect sizes measured in this study are small and only indicative of the direction of association between social media exposure and food intake, without determining the amount of food consumed in response to social media exposure. Accordingly, future studies could benefit from determining the effect of social media food messages and marketing on adolescent eating.

Despite these limitations, this study fills a gap in the literature and offers important insights to the field of health communication. In a sample exclusive to adolescents, we study how digital – specifically social media – food messages and marketing are associated with eating outcomes including food literacy, norms, attitude, intentions and intake. We introduce mediation models that provide a deeper insight in the underpinnings of the basic relation between exposure to messages and eating outcomes. In particular, we propose that food literacy and perceived norms mediate the relationship between social media exposure (to food messages) and adolescent eating. The study also benefits from a large sample of adolescents that included younger and older adolescents from different educational categories. Furthermore, this research measured reported exposure to all social media platforms and was not limited to one or two types of social media. On the contrary, respondents were introduced to the definition of social media and examples of common social media platforms such as Facebook, Instagram and YouTube, prior to answering questions about social media.

This study highlights important insights in the area of social media food marketing targeting adolescents, an age group that is generally neglected in the literature yet assumed by most policy makers to be resilient towards food marketing despite their profuse use of these media. Among a sample of 1002 adolescents, social media non-core food messages and marketing were positively associated with non-core food intake, preferences and perceived norms, as well as negatively associated with food literacy. This has important implications for policy makers in the provisions of regulations that control food marketing on social media. This study also demonstrated the importance of social media core food messages and food literacy in increasing core food eating among adolescents. As such, we recommend health professionals and brands marketing healthy foods to focus on social media messages that increase knowledge, attitude and skills that facilitate planning, choosing, preparing and consuming core food. This study also has important implications for future research. Firstly, we recommend research assessing the relationship between social media food exposure and adolescent eating to take into account the role of perceived norms and food literacy. Future research could closely examine how perceived norms and food literacy interplay in the relation between food messages and eating attitudes and behaviours. More research is needed to investigate the mediation models developed in this study, including the assessment of more extensive core and non-core food lists, the investigation of this mediation models among other age groups (e.g. young adults) and the use of experimental designs to determine the causal relationship between actual social media exposure and food consumption, as well as the mediating role of perceived norms and food literacy. Furthermore, future experimental research could quantify the relationships we observed in this survey and determine the quantity of food consumed as a result of exposure to social media food messages. Finally, we also call for further research to identify specific food marketing strategies that affect perceived norms, food preferences and eating among adolescents.

## Supplementary material

For supplementary material accompanying this paper visit https://doi.org/10.1017/S1368980021003116. click here to view supplementary material
