# Supplementary material for: Food for teens: how social media is associated with adolescent eating outcomes
Source: Public Health Nutr. 2021 Jul 30;25(2):290–302. doi: 10.1017/S1368980021003116 (PMC8883778; doi:10.1017/S1368980021003116)
Supplement: Supplementary file 1 [file S1368980021003116sup001.docx]

**Supplementary Material**

Table 1 An appraisal of how the survey data was reported using the JBI Critical Appraisal Checklist for Analytical Cross Sectional Studies

| **Criteria** | **Appraisal** |
| --- | --- |
| Were the criteria for inclusion in the sample clearly defined? | The inclusion and exclusion criteria were defined |
| Were the study subjects and the setting described in detail? | Study subjects and setting were described |
| Was the exposure measured in a valid and reliable way? | The independent variable was checked for face validity. The three researchers involved in data collection were trained to and followed a standard data collection procedure. |
| Were confounding factors identified? | Confounding variables were identified |
| Were strategies to deal with confounding factors stated? | Multivariate analysis was used to deal with confounding variables |
| Were the outcomes measured in a valid and reliable way? | The outcomes were measured using a validated Flemish Food Frequency Questionnaire. The three researchers involved in data collection were trained and followed a standard data collection procedure. |
| Was appropriate statistical analysis used? | Detailed information on the statistical analysis used in the study is provided |

Table 2 The measures used to assess major concepts in the survey and the sources they were retrieved/adapted from.

| Concept | Measure | Source |
| --- | --- | --- |
| Food messages exposure | 35 items inquiring about the extent to which the participant saw core and non-core food messages on their social media. | Developed and tested (cognitive interviews and pilot) by the authors for the purpose of this survey. |
| Food intake | Flemish food frequency questionnaire | Matthys C, Meulemans A, Van Der Schueren B (2015) Development and validation of general FFQ for use in clinical practice. *Annals of Nutrition and Metabolism* 67(Abstract No. 149/690):239. |
| Intention to eat | “Think about the next month. Compared to what you eat now, how much of these foods do you plan to eat? ” | Dixon HG, Scully ML, Wakefield MA, et al. (2007) The effects of television advertisements for junk food versus nutritious food on children's food attitudes and preferences *Soc Sci Med*;65(7):1311-23. |
| Food attitudes | “How much do you like each of these foods?”  “In your opinion, how healthy are each of these foods?” | Dixon HG, Scully ML, Wakefield MA, et al. (2007) The effects of television advertisements for junk food versus nutritious food on children's food attitudes and preferences *Soc Sci Med*;65(7):1311-23. |
| Perceived descriptive norms | “How often do you think other children your age consume this food?”. | Dixon HG, Scully ML, Wakefield MA, et al. (2007) The effects of television advertisements for junk food versus nutritious food on children's food attitudes and preferences *Soc Sci Med*;65(7):1311-23. |
| Perceived injunctive norms | “How healthy do others think this food is?” | Dixon HG, Scully ML, Wakefield MA, et al. (2007) The effects of television advertisements for junk food versus nutritious food on children's food attitudes and preferences *Soc Sci Med*;65(7):1311-23. |
| Food literacy | Self-perceived food literacy scale | Poelman MP, Dijkstra SC, Sponselee H, et al. (2018) Towards the measurement of food literacy with respect to healthy eating: the development and validation of the self perceived food literacy scale among an adult sample in the Netherlands. *Int J Behav Nutr Phys Act* 15(1):54. |
| Self-regulated autonomy | Treatment self-regulation questionnaire | Center for Self-Determination Theory (2021) Self-Regulation Questionnaire: <https://selfdeterminationtheory.org/self-regulation-questionnaires/>.] |

Table 3 Median, first and third quartile scores of reported exposure to food messages on social media among adolescents 12-19 years of age

| Variable | Median | Q1 | Q3 |
| --- | --- | --- | --- |
| Reported Exposure to Core food posts | 2.58 | 1.67 | 3.42 |
| Reported Exposure to Non-Core food posts | 4.08 | 3.17 | 5.00 |
| Reported Exposure to Branded non-Core food posts | 5.00 | 3.67 | 6.00 |

Table 4 Frequency of non-core and core food intake among a sample of 1002 Flemish adolescents 12-19 years old

| Frequency | Sweets  (N) | Soft drinks  (N) | Fried food  (N) | Vegetables  (N) | Fruit  (N) |
| --- | --- | --- | --- | --- | --- |
| Never | 22 | 52 | 17 | 25 | 40 |
| 1-3 days per month | 80 | 144 | 243 | 51 | 111 |
| 1 day per week | 170 | 163 | 454 | 65 | 128 |
| 2-4 days per week | 324 | 254 | 225 | 219 | 286 |
| 5-6 days per week | 148 | 101 | 43 | 219 | 149 |
| Daily | 254 | 286 | 9 | 418 | 285 |

Table 5 Non-core and core food intake among a sample of 1002 Flemish adolescents 12-19 years old

| Consumption per month | Median | QR1 | QR3 |
| --- | --- | --- | --- |
| Sweets (g) | 9.42 | 3.00 | 22.00 |
| Soft drinks (ml) | 125.00 | 53.57 | 375.00 |
| Vegetables (g) | 102.86 | 51.43 | 240.00 |
| Fruit (g) | 117.86 | 32.14 | 275.00 |

Table 6 Amount of water consumption per day among a sample of 1002 Flemish adolescents 12-19 years old

| Water intake  Amount per day | N |
| --- | --- |
| 500 ml or less | 213 |
| 501- 750 ml | 297 |
| 751 - 1250ml | 338 |
| more than 1250ml | 149 |

Table 7 Fit values before and after removal of self-regulated autonomy (TSR scores) and injunctive norms

| Non-core post models | | | | | |
| --- | --- | --- | --- | --- | --- |
|  | **Sweets (Freq)** | **Sweets (g/day)** | **Soft drinks (Freq)** | **Soft drinks (ml /day)** | **Fried food (Freq)** |
| CFI  Models with TSR & Injunctive norms  Models without TSR & Injunctive norms | 0.781-0.776    0.940 0.938 | 0.895 0.915  0.940 0.938 | 0.795 0.789  0.921 0.917 | 0.911  0.979 | 0.788 0.784  0.869 0.867 |
| SRMR  Models with TSR & Injunctive norms  Models without TSR & Injunctive norms | 0.056       0.056 | 0.027  0.026  0.034 | 0.048  .048  0.034 | 0.027  0.017 | 0.056  0.047 |
| RMSEA  Models with TSR & Injunctive norms  Models without TSR & Injunctive norms | 0.103       0.102 | 0.108  0.087 0.082 | 0.082 0.081  0.082 | 0.107  0.079 | 0.103 0.101  0.055 0.061 |
| Branded non-core post models | | | | | |
| CFI  Models with TSR & Injunctive norms  Models without TSR & Injunctive norms | 0.759  0.754  0.922 0.920 | 0.877  0.960 | 0.651 0.642  0.921  0.917 | 0.900  0.979 | 0.809  0.805  0.950       0.948 |
| SRMR  Models with TSR & Injunctive norms  Models without TSR & Injunctive norms | 0.109 0.108  0.038 | 0.029  0.019 | 0.060  0.034 | 0.028  0.017 | 0.057  0.033 |
| RMSEA  Models with TSR & Injunctive norms  Models without TSR & Injunctive norms | 0.060  0.094 | 0.116  0.096 | 0.109  0.108  0.082 | 0.112  0.079 | 0.103  0.102  0.082 |
| Core post models | | | | | |
|  | **Vegetables (Freq)** | **Vegetables (g/day)** | **Fruits (Freq)** | **Fruits (ml /day)** | **Water (ml/day)** |
| CFI  Models with TSR & Injunctive norms  Models without TSR & Injunctive norms | 0.703 0.693  0.987 | 0.826  0.984 | 0.413 0.398  1.000 | 0.355  0.521 | 0.413-0.398  0.965-0.964 |
| SRMR  Models with TSR & Injunctive norms  Models without TSR & Injunctive norms | 0.078  0.022 | 0.036  0.013 | 0.152  0.150  0.016 | 0.094  0.075 | 0.082  0.022 |
| RMSEA  Models with TSR & Injunctive norms  Models without TSR & Injunctive norms | 0.138 0.136  0.044 | 0.140  0.059 | 0.082  0.000 | 0.290  0.277 | 0.152 0.150  0.044 |

*CFI: , Comparative Fit Index, SRMR: Standardized Root Mean Square Residual, RMSEA : Root Mean Square Error of Approximation

Table 8 Mediation models of the relationship between reported exposure to non-core food posts on social media and reported non-core food intake among adolescents

|  | Sweets (Freq ) | | | Sweets (g/day) | | | Soft drinks (Freq) | | | Soft drinks (ml /day) | | | | Fried food (Freq) | | |
| --- | --- | --- | --- | --- | --- | --- | --- | --- | --- | --- | --- | --- | --- | --- | --- | --- |
|  | Estimate | SE | p.value | Estimate | SE | p.value | Estimate | SE | p.value | Estimate | SE | p.value | Estimate | | SE | p.value |
| Direct effects |  |  |  |  |  |  |  |  |  |  |  |  |  | |  |  |
| Non-core posts | 0.015 | 0.007 | **0.027** | 0.015 | 0.006 | **0.022** | 0.007 | 0.008 | 0.388 | 0.013 | 0.009 | 0.145 | 0.033 | | 0.007 | **0.000** |
| Indirect effect |  |  |  |  |  |  |  |  |  |  |  |  |  | |  |  |
| Descriptive  norms | 0.008 | 0.002 | **0.000** | 0.005 | 0.002 | **0.001** | 0.003 | 0.001 | **0.033** | 0.002 | 0.002 | 0.140 | 0.002 | | 0.001 | 0.065 |
| Injunctive  norms | -0.001 | 0.001 | 0.121 | -0.001 | 0.001 | 0.072 | -0.001 | 0.001 | 0.220 | -0.001 | 0.001 | 0.327 | 0.001 | | 0.001 | 0.113 |
| Food literacy | -0.000 | 0.002 | 0.800 | -0.001 | 0.002 | 0.725 | -0.000 | 0.002 | 0.800 | 0.001 | 0.002 | 0.755 | -0.001 | | 0.002 | 0.776 |
| Total indirect | 0.006 | 0.002 | **0.016** | 0.001 | 0.003 | 0.185 | **0.002** | 0.002 | 0.483 | 0.003 | 0.002 | 0.490 | 0.003 | | 0.003 | 0.257 |
| Total effect | 0.011 | 0.008 | 0.135 | 0.018 | 0.007 | 0.007 | 0.008 | 0.008 | 0.292 | 0.015 | 0.008 | 0.094 | 0.036 | | 0.008 | **0.000** |
| Covariates |  |  |  |  |  |  |  |  |  |  |  |  |  | |  |  |
| Age | -0.011 | 0.018 | 0.522 | -0.009 | 0.016 | 0.580 | 0.025 | 0.018 | 0.177 | 0.024 | 0.022 | 0.278 | 0.007 | | 0.019 | 0.727 |
| Gender | 0.080 | 0.079 | 0.308 | 0.058 | 0.068 | 0.395 | -0.210 | 0.084 | 0.013 | -0.488 | 0.094 | 0.000 | -0.156 | | 0.080 | 0.052 |
| BMI-for-age | -0.051 | 0.027 | 0.058 | -0.051 | 0.028 | 0.071 | 0.018 | 0.033 | 0.582 | 0.015 | 0.039 | 0.689 | 0.030 | | 0.034 | 0.378 |
| TSR | -0.009 | 0.026 | 0.712 | -0.013 | 0.025 | 0.588 | -0.055 | 0.027 | 0.038 | -0.071 | 0.034 | 0.037 | -0.062 | | 0.025 | 0.013 |

 * Gender: 0 male, 1 female

Table 9 Mediation models of the relationship between branded non-core food ad posts on social media and non-core food intake among adolescents

|  | **Sweets (Freq)** | | | **Sweets (g/day)** | | | **Soft drinks (Freq)** | | | **Soft drinks (ml /day)** | | | **Fried food (Freq)** | | |
| --- | --- | --- | --- | --- | --- | --- | --- | --- | --- | --- | --- | --- | --- | --- | --- |
|  | Estimate | SE | p.value | Estimate | SE | p.value | Estimate | SE | p.value | Estimate | SE | p.value | Estimate | SE | p.value |
| **Direct effects** |  |  |  |  |  |  |  |  |  |  |  |  |  |  |  |
| **Branded non-core posts** | 0.024 | 0.023 | 0.308 | 0.034 | 0.021 | 0.101 | -0.008 | 0.024 | 0.724 | 0.000 | 0.009 | 0.99 | 0.030 | 0.017 | 0.168 |
| **Indirect effect** |  |  |  |  |  |  |  |  |  |  |  |  |  |  |  |
| **Descriptive norms** | 0.023 | 0.006 | **0.000** | 0.017 | 0.005 | **0.001** | 0.009 | 0.004 | **0.023** | 0.010 | 0.006 | 0.081 | 0.010 | 0.004 | **0.014** |
| **Injunctive norms** | -0.000 | 0.002 | 0.760 | -0.001 | 0.002 | 0.727 | -0.000 | 0.001 | 0.763 | -0.001 | 0.001 | 0.557 | 0.000 | 0.002 | 0.878 |
| **Food literacy** | -0.006 | 0.005 | 0.216 | -0.008 | 0.006 | 0.191 | -0.006 | 0.005 | 0.218 | -0.009 | 0.010 | 0.369 | -0.009 | 0.007 | 0.223 |
| **Total indirect** | 0.016 | 0.008 | **0.043** | 0.009 | 0.008 | 0.270 | 0.003 | 0.007 | 0.668 | -0.000 | 0.011 | 0.998 | 0.001 | 0.008 | 0..868 |
| **Total effect** | 0.040 | 0.024 | 0.097 | 0.043 | 0.022 | **0.048** | -0.005 | 0.024 | 0.824 | 0.000 | 0.030 | 0.991 | 0.032 | 0.024 | 0.192 |
| **Covariates** |  |  |  |  |  |  |  |  |  |  |  |  |  |  |  |
| **Age** | -0.012 | 0.018 | 0.506 | -0.009 | 0.016 | 0.570 | 0.026 | 0.019 | 0.158 | 0.027 | 0.022 | 0.233 | 0.008 | 0.019 | 0.678 |
| **Gender** | 0.097 | 0.077 | 0.205 | 0.090 | 0.067 | 0.179 | -0.213 | 0.082 | **0.009** | -0.470 | 0.092 | **0.000** | -0.086 | 0.077 | 0.263 |
| **BMI-for-age** | -0.049 | 0.027 | 0.072 | -0.049 | 0.028 | 0.083 | 0.020 | 0.033 | 0.545 | 0.020 | 0.039 | 0.612 | 0.035 | 0.035 | 0.314 |
| **TSR** | -0.012 | 0.025 | 0.636 | -0.016 | 0.024 | 0.523 | -0.055 | 0.027 | **0.040** | -0.074 | 0.034 | **0.029** | -0.070 | 0.024 | **0.004** |

* Gender: 0 male, 1 female

Table 10 Mediation models of the relationship between core food posts on social media and core food intake among adolescents

|  | **Vegetables (Freq)** | | | **Vegetables (g/day)** | | | **Fruits (Freq)** | | | **Fruits (ml /day)** | | | **Water (ml/day)** | | | |
| --- | --- | --- | --- | --- | --- | --- | --- | --- | --- | --- | --- | --- | --- | --- | --- | --- |
|  | Estimate | SE | p.value | Estimate | SE | p.value | Estimate | SE | p.value | Estimate | SE | p.value | Estimate | SE | p.value |  |
| **Direct effects** |  |  |  |  |  |  |  |  |  |  |  |  |  |  |  |  |
| **Core posts** | -0.011 | 0.009 | 0.219 | 0.011 | 0.009 | 0.226 | 0.005 | 0.008 | 0.548 | 0.040 | 0.043 | 0.359 | 0.006 | 0.009 | 0.489 |  |
| **Indirect effect** |  |  |  |  |  |  |  |  |  |  |  |  |  |  |  |  |
| **Descriptive norms** | 0.002 | 0.001 | 0.100 | 0.002 | 0.001 | 0.072 | -0.000 | 0.001 | 0.841 | 0.006 | 0.005 | 0.221 | -0.002 | 0.001 | **0.122** |  |
| **Injunctive norms** | -0.000 | 0.000 | 0.399 | -0.000 | 0.001 | 0.447 | -0.000 | 0.000 | 0.796 | -0.002 | 0.004 | 0.575 | 0.000 | 0.000 | 0.656 |  |
| **Food literacy** | 0.010 | 0.003 | **0.000** | 0.013 | 0.003 | **0.000** | 0.009 | 0.002 | **0.000** | 0.068 | 0.018 | **0.000** | 0.008 | 0.002 | **0.000** |  |
| **Total indirect** | 0.012 | 0.003 | **0.000** | 0.015 | 0.004 | **0.000** | 0.008 | 0.003 | **0.002** | 0.071 | 0.019 | **0.000** | 0.006 | 0.002 | **0.015** |  |
| **Total effect** | 0.000 | 0.010 | 0.974 | 0.026 | 0.010 | **0.007** | 0.013 | 0.009 | 0.125 | 0.111 | 0.047 | **0.018** | 0.012 | 0.009 | 0.173 |  |
| **Covariates** |  |  |  |  |  |  |  |  |  |  |  |  |  |  |  |  |
| **Age** | -0.003 | 0.019 | 0.865 | -0.033 | 0.020 | 0.099 | -0.067 | 0.019 | **0.001** | -0.509 | 0.095 | **0.000** | -0.012 | 0.020 | 0.537 |  |
| **Gender** | 0.078 | 0.082 | 0.344 | -0.013 | 0.084 | 0.875 | -0.026 | 0.081 | 0.745 | -0.402 | 0.395 | 0.310 | -0.396 | 0.079 | **0.000** |  |
| **BMI-for-age** | -0.088 | 0.030 | **0.007** | -0.039 | 0.028 | 0.083 | -0.059 | 0.032 | 0.066 | -0.025 | 0.163 | 0.879 | 0.119 | 0.029 | **0.000** |  |
| **TSR** | 0.002 | 0.027 | 0.937 | -0.005 | 0.031 | 0.881 | -0.016 | 0.029 | 0.585 | -0.140 | 0.144 | 0.332 | 0.008 | 0.026 | 0.751 |  |

* Gender: 0 male, 1 female
